# Effect of infection with hepatitis B virus on the survival outcome of diffuse large B-cell lymphoma in the prophylactic antiviral era

**DOI:** 10.3389/fonc.2022.989258

**Published:** 2022-08-22

**Authors:** Reyizha Nuersulitan, Miaomiao Li, Lan Mi, Meng Wu, Xinqiang Ji, Yiqi Liu, Hong Zhao, Guiqiang Wang, Yuqin Song, Jun Zhu, Weiping Liu

**Affiliations:** ^1^ Key Laboratory of Carcinogenesis and Translational Research (Ministry of Education), Department of Lymphoma, Peking University Cancer Hospital & Institute, Beijing, China; ^2^ Key Laboratory of Carcinogenesis and Translational Research (Ministry of Education), Department of Medical Record Statistics, Peking University Cancer Hospital & Institute, Beijing, China; ^3^ Department of Infectious Diseases, Peking University First Hospital, Beijing, China

**Keywords:** hepatitis B virus, infections, survival, lymphoma, large B-cell, diffuse, antiviral agents

## Abstract

Patients with lymphoma who are also infected with Hepatitis B virus (HBV) have a poor prognosis. This could be partly explained by the delay or premature termination of anti-tumor treatment because of HBV reactivation. However, there is limited data on the survival outcome of patients HBV-related lymphoma in the era of prophylactic antivirals. Data for 128 patients with HBV surface antigen-positive diffuse large B-cell lymphoma was collected. The median age was 54 years and the ratio of men to women was 1.2:1. All patients received immune-chemotherapy and prophylactic antiviral therapy. The median number of cycles of immune-chemotherapy was six. The overall response rate was 82%, with a complete remission rate of 75%. With a median follow-up of 58.4 months, the 5-year progression-free survival and overall survival rates were 75.7% and 74.7%, respectively. Nine patients experienced HBV reactivation but none experienced HBV-associated hepatitis. Patients with low and high HBV DNA loads had comparable survival outcomes. In conclusion, HBV infection had no negative effect on the prognosis of DLBCL in the era of prophylactic antiviral therapy.

## Introduction

In the last 30 years in China, the lymphoma disease burden has increased gradually (1). One of the most common types of non-Hodgkin lymphoma (NHL) is diffuse large B-cell lymphoma (DLBCL), representing nearly 30–40% of cases (2, 3). The survival outcome of DLBCL has improved because of iterated treatment strategies. About 60–70% of patients with DLBCL can be cured using immunochemotherapy with rituximab, prednisone, vincristine, doxorubicin, and cyclophosphamide (the R-CHOP) regimen. A retrospective study involving 1633 patients with DLBCL showed that the 5-year overall survival (OS) increased from 47.2% in 1995–1999 to 63.4% in 2011–2015, and those treated with rituximab and anthracyclines had a superior 5-year OS compared with those treated with chemotherapy alone (69.0% *vs.* 51.7%) (4).

The risk of developing NHL is 2–3 fold higher in patients with hepatitis B virus (HBV) infection, especially for DLBCL (5–7). In addition, patients with HBV infection tend to experience HBV reactivation during antitumor therapy. Both anti-CD20 monoclonal antibodies and anthracycline therapy have a high (> 10%) risk of HBV reactivation (8, 9). Approximately 20–50% of patients who are positive for hepatitis B surface antigen (HBsAg) and 3–45% of patients who are positive for hepatitis B core antibody (HBcAb) experienced HBV reactivation if anti-HBV therapy is not used (10–14). Notably, HBV reactivation might cause acute hepatitis, which interrupts antitumor therapy and has a negative impact on prognosis.

However, limited data on the survival outcome of patients with lymphoma and HBV infection is available for the period when prophylactic anti-HBV therapy started to be widely used. This study aimed to reveal the effect of infection with HBV on the survival outcome of DLBCL in the prophylactic antivirus therapy era.

## Methods

### Patients

This retrospective study comprised a review of the clinical data of patients diagnosed between 1^st^ January 2007 and 30^st^ June 2021 at Peking University Cancer Hospital. The key inclusion criteria included (1) HBsAg-positive (2); confirmed diagnosis of DLBCL using a biopsy specimen (3); receipt of at least four cycles of R-CHOP immunochemotherapy; and (4) treated with prophylactic antivirals. The exclusion criteria comprised (1) involvement of the central nervous system; and (2) Human immunodeficiency virus or Hepatitis C virus coinfection. To evaluate the effect of infection with HBV on the prognosis of DLBCL, we chose a control group who were not infected with HBV (negative for both HBsAg and HBcAb).

### Data collection

Medical records and charts provided the patient data, which included age, sex, diagnosis, Eastern Cooperative Oncology Group (ECOG) status, International Prognostic Index (IPI) score, stage, pathology, immunohistochemistry, laboratory tests, antiviral treatments, and the dose of the regimen of each cycle. We tested liver function (alanine aminotransferase [ALT]) and viral load (HBV DNA) at baseline. Then, HBV DNA and ALT levels were tested every 3 weeks during chemotherapy and every 3 months within 1 year after the cessation of chemotherapy.

### Definitions

The overall response rate (ORR) was defined as the proportion of patients who achieving either a partial response (PR) or a confirmed complete response (CR). Progression-free survival (PFS) was defined as the time from the date of diagnosis of DLBCL to the date of progression or death. Overall survival (OS) was defined as the time from the date of diagnosis of DLBCL to the date of death or last follow-up. A low HBV DNA load was defined as an HBV DNA level < 2000 international units (IU)/mL, and an HBV DNA level ≥ 2000 IU/mL defined a high HBV DNA load. HBV DNA was monitored from the start of treatment until 12 months after the final chemotherapy course. HBV reactivation was defined as: 1) HBV DNA increased by 100-fold or above if the baseline HBV DNA was detectable; 2) HBV DNA became detectable and more than 100 IU/mL if the baseline HBV DNA level was undetectable. HBV-associated hepatitis was defined as an ALT level ≥ 3-fold more than the upper limit of normal or was beyond 100 U/L concurrent with HBV reactivation (15). The cell-of-origin (COO) subtypes of DLBCL were determined based on the Hans algorithm (16).

### Treatment and response evaluation

For patients whose HBV DNA load was low, rituximab was administered from the first cycle of anti-tumor therapy. For patients whose HBV DNA load was high, rituximab was initiated when the HBV DNA decreased to 2000 IU/mL or below. We started the patients on prophylactic antiviral treatment from the start of antitumor therapy and continued it for at least 1 year after completion of the last cycle of antitumor therapy. Computed tomography (CT) or positron emission tomography (PET)/CT were used to evaluate staging and responses.

### Statistical analysis

The Kaplan–Meier method was used to determine the probability of PFS and OS and were analyzed employing a log-rank test. According to the IPI risk groups, a 1:2 propensity score-matched (PSM) analysis was carried out employing the nearest-neighbor method (caliper size = 0.02) to compare the survival outcomes of patients with DLBCL with different HBV infection statuses. A χ^2^ test was used to compare all the categorical variables after propensity score matching. The IBM Statistics SPSS 26.0 (IBM Corp. Armonk, NY, USA) and R version 4.1.3 (2022–03–10), (https://www.R-project.org/) were used to carry out the statistical analyses.

## Results

### Clinical characteristics

A total of 3521 patients were screened and 128 HBsAg-positive patients were included. The patients’ baseline clinical characteristics are shown in **Table 1**. The patients’ median age was 54 years and the male/female ratio was 1.2:1. Seventy-two (56.3%) patients suffered from advanced-stage disease (stage III/IV), and 18 (14.1%) had a high IPI score. A total of 122 patients had available data about the cell of origin, of which 77 (63.1%) were from the germinal center (GCB) and 45 (36.9%) were from the non-germinal center (non-GCB). Ninety-five (74.2%) patients’ HBV DNA load was low (< 2000 IU/mL) and 33 (25.8%) had a high load of HBV DNA (≥ 2000 IU/mL). One patient had a high ALT level (241 IU/L) at baseline, and received immunochemotherapy when ALT decreased to 100 IU/L after anti-inflammatory treatment. Due to the nature of high barrier to resistance, entecavir was used in our center since 2013. A total of 109 (85.2%) patients received entecavir therapy, 17 (13.3%) patients received lamivudine therapy, and 2 patients (1.5%) received adefovir therapy. Seventeen patients received antiviral treatment for chronic hepatitis B rather than prophylaxis purpose. None had decompensated liver cirrhosis.

**Table 1:** Patients with hepatitis B virus surface antigen (HBsAg)-positive diffuse large B-cell lymphoma clinical characteristics (N = 128).

Characteristics	No. of patients (%)
**Sex**	
Male	71 (55.5)
Female	57 (44.5)
**Age (years)**	
≥ 60	46 (35.9)
<60	82 (64.1)
** Stage**	
I/II	56 (43.8)
III/IV	72 (56.2)
** B symptoms**	
Yes	42 (32.8)
No	86 (67.2)
**ECOG Score**	
ECOG ≥ 2	7 (5.5)
ECOG < 2	121 (94.5)
**Cell of Origin**	
Non-GCB	77 (60.2)
GCB	45 (35.2)
NA	6 (4.6)
**IPI Score**	
0–1	68 (53.1)
2	28 (21.9)
3	18 (14.1)
4–5	14 (10.9)
**LDH**	
Elevated	54(42.2)
Normal	74 (57.8)
**Serological HBV markers**	
HBsAg (+) HBeAg (+) HBcAb (+)	19 (14.8)
HBsAg (+) HBeAb (+) HBcAb (+)	92 (71.9)
**HBV reactivation**	
Occurred	9 (7.0)
**Prophylactic treatments**	
Entecavir	109(85.2)
Lamivudine	17(13.3)
Others	2(1.5)

ECOG, Eastern Cooperative Oncology Group; GCB, germinal center B-cell-like; NA, not available; IPI, International Prognostic Index; LDH, lactate dehydrogenase; HBV, Hepatitis B virus; IU, international unit; HBsAg, Hepatitis B surface antigen; HBeAg, Hepatitis B e antigen; HBeAb, Hepatitis B e antibody; HBcAb, Hepatitis B core antibody.

### Treatments and responses

Rituximab was administered using a median of six cycles (range, 2–8) and chemotherapy was also administered using a median of six cycles (range, 4–8). Twenty-seven patients delayed the use of Rituximab because of a high HBV DNA load. Thirty patients did not receive prednisone because of a high HBV DNA load (n = 16) or because of gastrointestinal involvement (n = 14). In total, 96 patients achieved CR (75.0%), 9 achieved PR (7.0%), 1 achieved SD (0.8%), and 22 experienced PD (17.2%). The response rates were comparable between patients in the low and high HBV DNA load groups (ORR, 84.8% *vs*. 80.0%, *P* = 0.54; CR rate, 72.7% *vs*. 74.7%, *P*=0.82; respectively).

### HBV-related events

The median number of times HBV DNA was tested was 7 and the median number of times ALT was tested was 9 for each patient from the start of anti-tumor treatment to 1 year after the cessation of immunochemotherapy. HBV reactivation was observed in nine (7.0%) patients during immunochemotherapy, and was not observed after the cessation of immunochemotherapy **(** **Table S1** **)**. Two patients had positive hepatitis B e antigen. In terms of antiviral drug, 8 out of 109 (7.3%) patients treated with entecavir and 1 out of 17 (5.9%) patients treated with lamivudine experienced HBV reactivation. The median interval from the start of chemotherapy to HBV reactivation was 1.5 months (range: 1–4 months). The dynamic changes in HBV DNA levels in patients with HBV reactivation are shown in **Figure S1**. However, HBV-associated hepatitis was not observed in any of these nine patients. Four patients had delayed scheduled immunochemotherapy and one had premature termination of rituximab.

### Survival analysis

**Figure 1:**
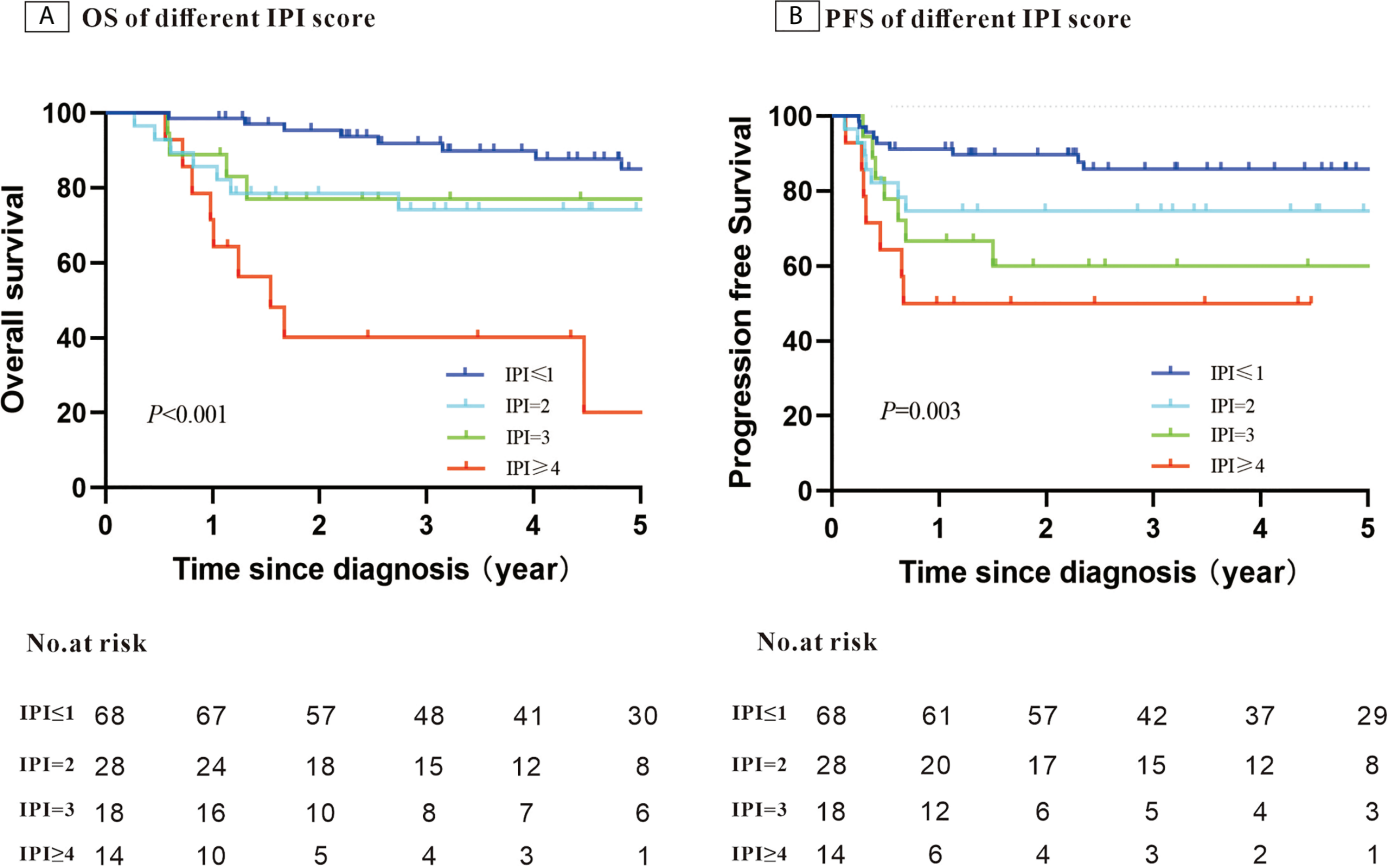
Kaplan–Meier survival curves for patients with Hepatitis B surface antigen (HBsAg)-positive diffuse large B-cell lymphoma (DLBCL) stratified using the patients’ International Prognostic Index (IPI) score (N = 128).

**Figure 2:**
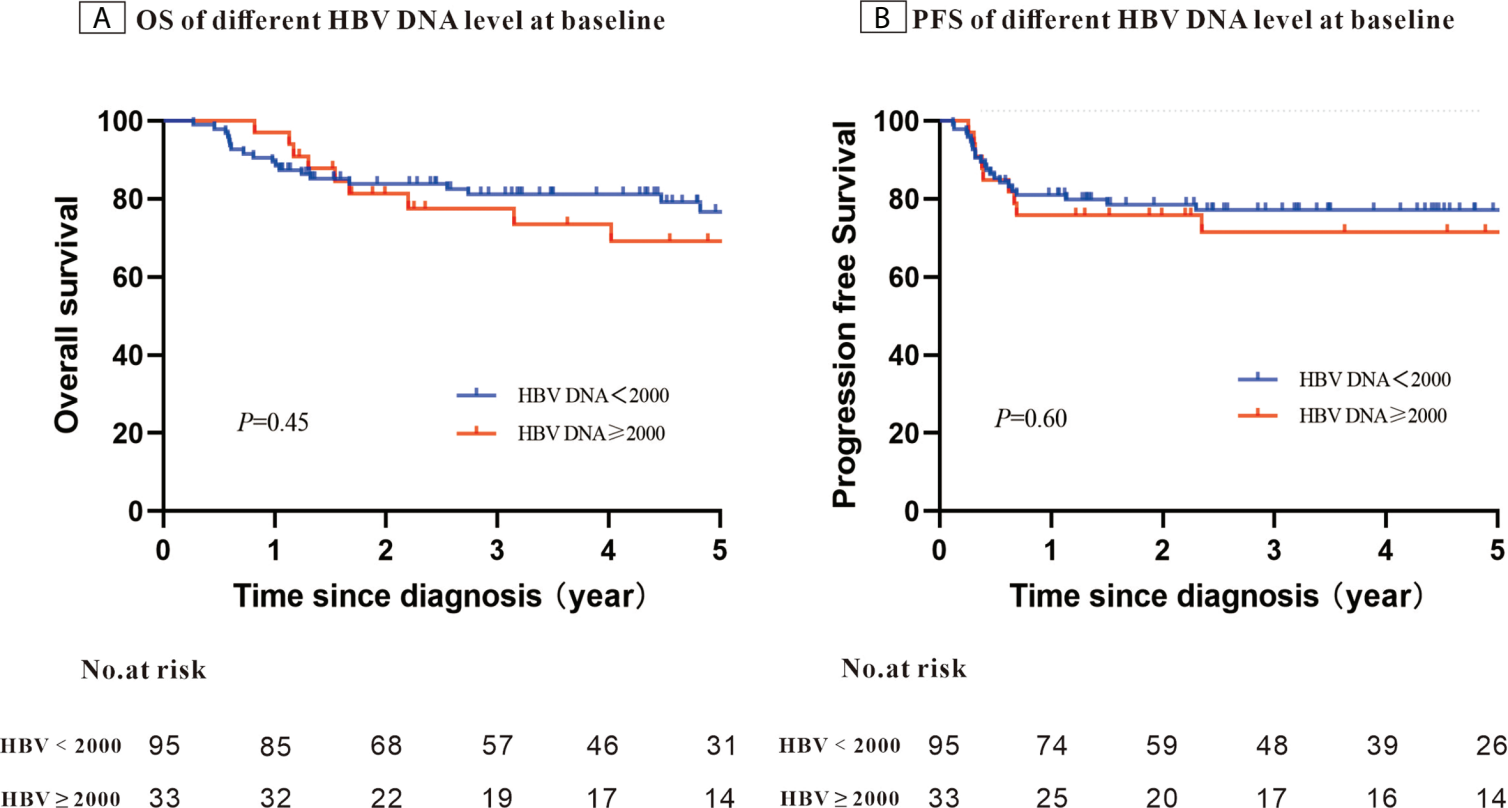
Kaplan–Meier survival curves for patients with Hepatitis B surface antigen (HBsAg)-positive diffuse large B-cell lymphoma (DLBCL) stratified using the patients’ Hepatitis B virus (HBV) DNA level at baseline (N = 128).

**Figure 3:**
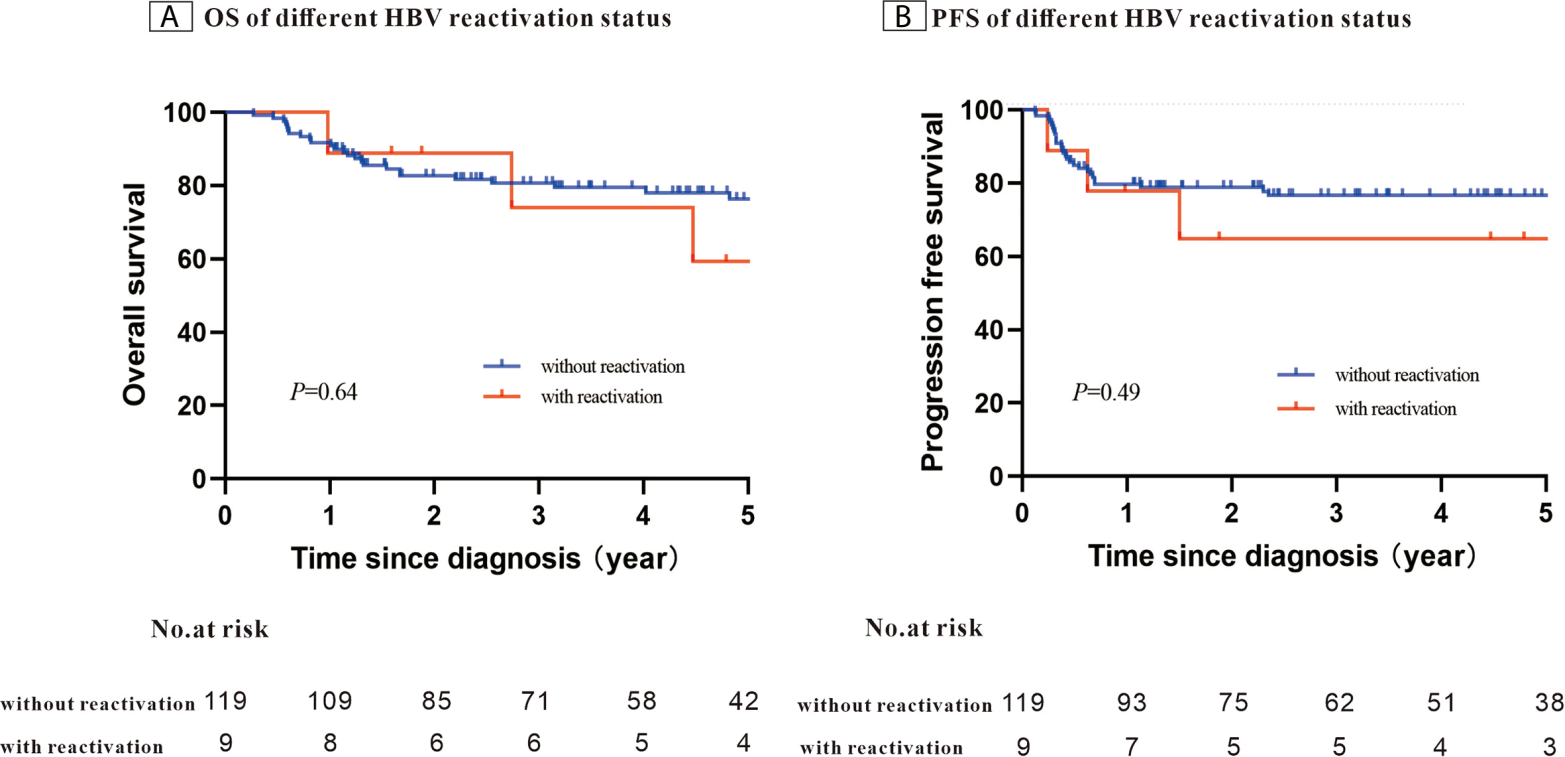
Kaplan–Meier survival curves for patients with Hepatitis B surface antigen (HBsAg)-positive diffuse large B-cell lymphoma (DLBCL) stratified using the patients’ Hepatitis B virus (HBV) reactivation status (N = 128).

After a median follow-up of 58.4 months, the 5-year PFS was 75.7% and OS was 74.7%. When stratifying patients into four groups according to their IPI scores, both PFS and OS differed significantly (OS: *P <* 0.001; PFS: *P* = 0.003) (**Figures 1A, B**). The 5-year OS and 5-year PFS rates were comparable between patients whose HBV DNA loads were high and those whose loads were low (5-year OS: 76.7% *vs*. 69.1%, *P* = 0.45; 5-year-PFS: 77.2% *vs*. 71.5%, *P*=0.60) (**Figures 2A, B**). Those patients with HBV reactivation had similar OS and PFS values compared with patients without HBV reactivation (5-year OS: 76.3% *vs*. 59.2%, *P* = 0.64; 5-year PFS: 76.6% *vs*. 64.8%, *P* = 0.46) (**Figures 3A, B**).

### Evaluation of effect of infection with HBV on patient survival

**Figure 4:**
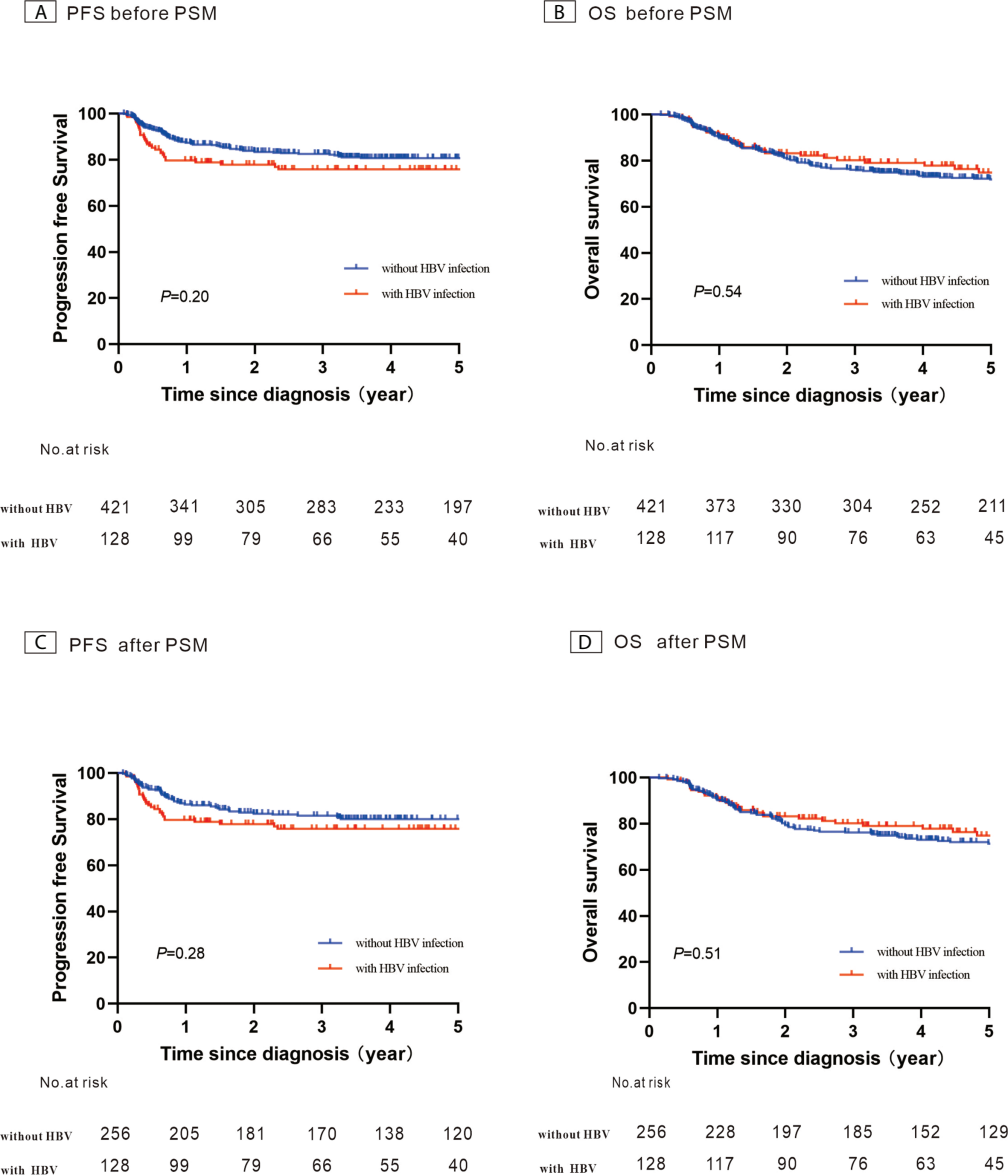
Kaplan–Meier survival curves for patients with diffuse large B-cell lymphoma constructed using their hepatitis B virus (HBV) infection status. **(A)** Progression-free survival (PFS) before propensity score-matched (PSM) analysis; **(B)** Overall survival (OS) before PSM analysis; **(C)** PFS after PSM analysis; **(D)** OS after PSM analysis.

A total of 421 newly diagnosed patients with DLBCL without HBV infection were chosen as the control group (**Table 2** **)**. Before PSM, the 5-year PFS and 5-year OS rates were 80.7% and 71.8% for the control group, respectively (**Figures 4A, B**). After 1:2 patients matching according to IPI risk scores, the 5-year PFS and 5-year OS rates were similar in the patients with HBV infection and in those without HBV infection (5-year PFS: 75.7% *vs*. 80.0%, *P* = 0.28; 5-year OS: 74.7% *vs*. 71.4%, *P* = 0.51) (**Figures 4C, D** **)**.

**Table 2:** Baseline characteristics of patients with and without HBV infection before and after Propensity Score Matching.

		Overall cohort			Matched cohort		
Characteristic		Without HBV infection	With HBV infection	*p*-Value	Without HBV infection	With HBV infection	*p*-Value
		(n = 421)	(n = 128)		(n = 256)	(n = 128)
**Sex**							
Female		222 (52.7)	57 (44.5)	0.104	128 (50.0)	57 (44.5)	0.608
Male		199 (47.3)	71 (55.5)		128 (50.0)	71 (55.5)	
**Age**							
< 60		257 (61.0)	82 (64.1)	0.538	152 (59.4)	82 (64.1)	0.375
≥ 60		164 (39.0)	46 (35.9)		104 (40.6)	46 (35.9)	
**Stage**							
I/II		217 (51.5)	56 (43.8)	0.122	133 (52.0)	56 (43.8)	0.130
III/IV		204 (48.5)	72 (56.2)		123 (48.0)	72 (56.2)	
**B symptoms**							
Yes		130 (30.9)	42 (32.8)	0.68	3 (5.36)	89 (34.8)	0.70
No		291 (69.1)	86 (67.2)		53 (94.64)	167 (65.2)	
**ECOG**							
ECOG ≥ 2		30 (7.1)	7 (5.5)	0.513	19 (7.4)	7 (5.5)	0.473
ECOG < 2		391 (92.9)	121 (94.5)		237 (92.6)	121 (94.5)	
**Cell of Origin**							
Non-GCB		246 (58.4)	77 (60.2)	0.127	148 (57.8)	77 (60.2)	0.096
GCB		131 (31.1)	45 (35.2)		79 (30.9)	45 (35.2)	
NA		44 (10.5)	6 (4.6)		29 (11.3)	6 (4.6)	
**IPI Score**							
0–1		231 (54.9)	68 (53.1)	0.231	136 (53.1)	68 (53.1)	1.000
2		66 (15.6)	28 (21.9)		56 (21.9)	28 (21.9)	
3		84 (20.0)	18 (14.1)		36 (14.1)	18 (14.1)	
4–5		40 (9.5)	14 (10.9)		28 (10.9)	14 (10.9)	
**LDH**							
Elevated		167 (39.7)	54 (42.2)	0.611	99 (38.7)	54 (42.2)	0.507
Normal		254 (60.3)	74 (57.8)		157 (61.3)	74 (57.8)	

ECOG, Eastern Cooperative Oncology Group; GCB, germinal center B-cell-like; NA, not available; IPI, International Prognostic Index; LDH, lactate dehydrogenase.

## Discussion

The present study comprehensively analyzed the survival outcomes of patients with DLBCL who were infected with HBV in the era of prophylactic antiviral therapy and evaluated the effect of HBV infection on patient prognosis using a case-case study method. We found that the survival outcome was comparable between patients with high and low HBV DNA loads. We also determined that HBV infection had no negative effect on the prognosis of patients with DLBCL when prophylactic antiviral therapy was used. Our study proved the evidence that patients with DLBCL with HBV infection could achieve a good outcome if they receive a standard anti-tumor therapy in the era of prophylactic antiviral therapy.

A high load of HBV DNA was related to HBV reactivation in the pre-prophylactic antiviral therapy era. A study (17) evaluated the incidence of HBV reactivation-associated hepatitis in 137 patients who received autologous hematopoietic stem cell transplantation but did not receive preemptive antiviral therapy. HBV reactivation-associated hepatitis was observed in 11 of 23 patients with HBsAg positivity and in 2 of 114 patients who were HBsAg-negative. Moreover, detectable HBV DNA led to an increase of 9.35-fold in the risk of HBV reactivation-associated hepatitis. However, the incidences of HBV reactivation and HBV-associated hepatitis have decreased significantly since prophylactic antiviral therapy was used widely. A randomized phase 3 study (18) confirmed that entecavir reduced the prevalence of HBV reactivation (6.6% *vs*. 30%) and HBV-associated hepatitis (0% *vs*. 13.3%) compared with lamivudine. In a retrospective study (19) involving 340 patients with B cell lymphoma (BCL) who were infected with HBV, the prevalence of HBV reactivation was lower in HBsAg-positive patients who were administered with antiviral prophylaxis compared with that in patients who were not (22.9% *vs*. 59.1%). In addition, entecavir prophylaxis was superior to lamivudine prophylaxis in terms of reducing the occurrence of HBV reactivation (6.3% *vs*. 39.3%). In the present study, only 6.3% of patients experienced HBV reactivation without severe hepatitis, which was partly explained by fact that 85% of these patients received entecavir prophylaxis. Although the HBV reactivation rates between entecavir and lamivudine had not statistically significant difference, it could be attributed to the fact that only 17 patients were treated with lamivudine in the present study. These findings supported the view that antiviral drugs with high efficiency and a low resistance barrier, such as entecavir, reduced the prevalence of HBV reactivation and HBV-associated hepatitis, and were suitable as prophylactic antivirals for patients with lymphoma and HBV infection who need to go through immunochemotherapy.

Based on the published guidelines (20–22) , the monitoring of HBV DNA and treatment with antiviral prophylaxis should be continued for at least 6–12 months after the last dose of anticancer treatment. HBV DNA should be tested monthly during treatment, and then every 3 months after cessation of prophylaxis antivirals. A retrospective study (23) involved 107 patients with DLBCL and HBV infection and demonstrated that none experienced HBV reactivation or HBV-related hepatitis during the prophylactic antiviral therapy. However, 10 (21.7%) of the 46 patients with positive HBsAg experienced delayed HBV reactivation when antiviral therapy was withdrawn. In the present study, all HBV reactivation occurred during immunochemotherapy rather than after the cessation of immunochemotherapy when all patients continued antiviral treatments for at least 1 year. These findings highlighted the need to determine the optimal course of antiviral therapy and the precise time of antiviral therapy withdrawal.

Correspondingly, patients with DLBCL and active HBV infection had similar outcomes to uninfected patients when they received immunochemotherapy and antivirals. A population-based study (24) indicated those patients with DLBCL and HBV infection had comparable survival outcome than those without HBV infection (median OS, not reached *vs.* 74.23 months, respectively) when antiviral prophylaxis was used. Another study (25) compared the treatment responses and survival between 116 patients with HBsAg-positive DLBCL and 278 patients with HBsAg-negative DLBCL, all of whom received rituximab-based therapy. The HBsAg-positive patients had similar ORR (97.4% versus 92.5%) and CR rate (89.7% versus 83.8%) to the HBsAg-negative patients. Furthermore, the two groups had a similar 4-year PFS (66.8% versus 73.7%) and OS (77.5% versus 82.2%). In the present study, we also observed that HBV-infected patients had comparable 5-year PFS and 5-year OS rates to those without HBV infection when they received adequate antitumor therapy. These findings determined that active HBV infection had no negative effect on the prognosis of patients with DLBCL and supported the use of standardized immunochemotherapy in the prophylactic antiviral era.

### Limitations

First, we did not conduct a central pathological review because of 13-year span. Second, due to the limited sample size, the effect of reducing rituximab on patient prognosis in the high HBV DNA load group was not evaluated. Third, dynamics of HBV DNA levels were not analyzed.

## Conclusions

HBV infection did not have a negative effect on the prognosis of patients with DLBCL when they were treated with immunochemotherapy in era of prophylactic antiviral therapy. Although antiviral therapy prophylaxis with high efficiency and low resistance barrier prevented the reactivation of HBV and hepatitis associated with HBV, more than 5% patients still experienced HBV reactivation. Thus, it is vitally important to monitor HBV DNA and ALT levels closely.

## Data availability statement

The raw data supporting the conclusions of this article will be made available by the authors, without undue reservation.

## Ethics statement

The ethical standards dictated by the institutional committee of Peking University Cancer Hospital were followed when carrying out all procedures involving human participants, which conformed with the tenets of the 1964 Declaration of Helsinki, its later amendments, or comparable ethical standards. Signed informed consent was provided by each enrolled patient.

## Author contributions

RN conceived and designed the study, analyzed the data, and drafted and revised the manuscript. ML, LM, MW, and XJ prepared and analyzed the data. YL, HZ, GW, and YS drafted and revised the manuscript. JZ and WL designed the study, interpreted the results, and drafted and revised the manuscript. All the authors have provided critical comments on the manuscript.

## Funding

This study was supported by the Capital’s Funds for Health Improvement and Research (Grant No. 2022-4-2156) and Clinical Research Fund for Distinguished Young Scholars of Beijing Cancer Hospital (Grant No. QNJJ202106).

## Acknowledgments

The authors thank all of the participating patients, pathologists, and statisticians for their invaluable contributions to this study.

## Conflict of interest

The authors declare that the research was conducted in the absence of any commercial or financial relationships that could be construed as a potential conflict of interest.

## Publisher’s note

All claims expressed in this article are solely those of the authors and do not necessarily represent those of their affiliated organizations, or those of the publisher, the editors and the reviewers. Any product that may be evaluated in this article, or claim that may be made by its manufacturer, is not guaranteed or endorsed by the publisher.
