# Supplementary material for: Effect of infection with hepatitis B virus on the survival outcome of diffuse large B-cell lymphoma in the prophylactic antiviral era
Source: Front Oncol. 2022 Aug 22;12:989258. doi: 10.3389/fonc.2022.989258 (PMC9441704; doi:10.3389/fonc.2022.989258)

Table S1. Clinical characteristics at HBV reactivation (n=9)

Patients No.	Age	Sex	Baseline						Regimen (Before HBV reactivation)	Antiviral Prophylaxis	At diagnosis of HBV reactivation				Clinical course
			Stage	IPI	Sero-logy*	ALT/AST	TBIL/DBIL	HBV DNA (IU/mL)			ALT/AST	TBIL/DBIL	HBV DNA (IU/mL)	Time of Reactivation (from date of 1st cycle of chemotherapy)	
1	63	M	4	3	3	16/29	6.8/2.4	22400	R-CHOP	Entecavir	6/14	9.1/2	2620000	4months	Alive
2	43	F	4	2	3	14/18	12.6/3.2	< 10	R-CHOP	Entecavir	26/21	8.2/2.3	607	2 months	Alive
3	49	M	2	1	1	33/38	8.7/3	< 10	R-CHOP	Entecavir	62/50	6.9/2.4	149	1.5 months	Alive
4	49	M	4	2	3	10/23	14.5/4.1	< 10	R-CHOP	Entecavir	9/11	10.4/3	7230	1 month	Died
5	68	M	4	4	1	18/23	13.2/4.0	< 10	R-CHOP	Entecavir	25/42	5.9/2.5	129	2 months	Died
6	42	M	3	3	2	47/40	6/4.2	< 10	R-CHOP	Entecavir	17/23	10.9/2.4	139	2 months	Alive
7	37	M	4	1	2	241/108	22.4/6.3	98.1	R-CHOP	Entecavir	16/27	18.8/4.8	26100	1 month	Alive
8	33	F	1	3	2	17/25	7.9/2.7	< 10	R-CHOP	Lamivudine	15/16	11.7/3.1	888	1.5 months	Alive
9	62	F	4	4	2	16/20	19.3/5.6	< 10	R-CHOP	Entecavir	16/31	19.3/5.3	131	1 month	Died

Serology *：1 refers to HBsAg（+）HBeAg（+）HBcAb(+), 2 refers to HBsAg（+）HBeAb（+）HBcAb(+) and 3 refers to the other types.

Figure S1. The dynamic changes of HBV DNA of patients occurred with HBV reactivation (n=9).

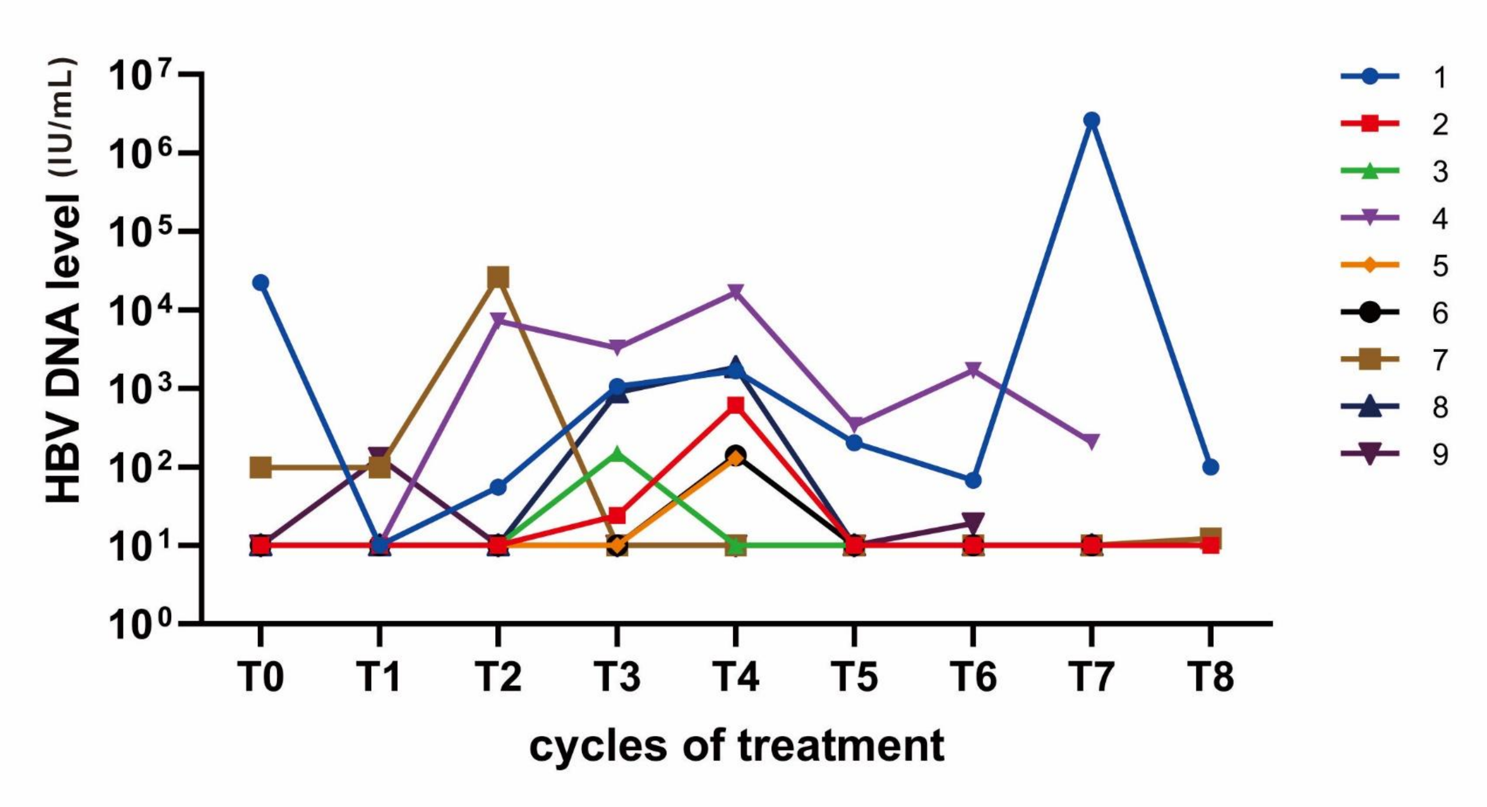

Supplement: Supplementary file 1 [file DataSheet_1.pdf]
